# Multifocal hemosiderin depositions on T2*-weighted magnetic resonance imaging in a patient with pathology-proven systemic diffuse large B-cell lymphoma

**DOI:** 10.1186/s12883-014-0184-1

**Published:** 2014-09-25

**Authors:** Xun-zhe Yang, Jun Ni, Li-ying Cui

**Affiliations:** Department of Neurology, Peking Union Medical College Hospital, Beijing, 100730 China

**Keywords:** Systemic lymphoma, Central nervous system, Hemosiderin deposition, T2*-weighted gradient-echo magnetic resonance imaging

## Abstract

**Background:**

Intracranial hemorrhage in central nervous system lymphoma is extremely rare. T2*-weighted gradient-echo magnetic resonance imaging is of particularly use in detecting silent hemorrhage as hypointense signals due to the deposition of paramagnetic hemosiderin or mineralization. Multifocal hemosiderin depositions caused by chronic silent hemorrhage have not yet been identified in patients with central nervous system involvement of systemic lymphoma. We present an unexpected radiographic feature on T2*-weighted gradient-echo magnetic resonance imaging in a patient with central nervous system involvement of pathologically confirmed systemic diffuse large B-cell lymphoma.

**Case presentation:**

A 56-year-old woman presented with lower extremities weakness and progressive cognitive decline for four months. Conventional brain magnetic resonance imaging demonstrated multiple lesions with hypointensities on T1-weighted images and hyperintensities on T2-weighted images and fluid attenuated inversion recovery in both hemispheres. She was then transferred to our hospital.

Neurological examination showed impaired cognitive functions. Contrast-enhanced magnetic resonance imaging revealed irregular spotty enhancement within the lesions. T2*-weighted gradient-echo magnetic resonance imaging revealed diffuse hemosiderin depositions with hypointensities mostly adjacent to the cortex, which are not compatible with the lesions visualized on T1-weighted images. Whole body positron emission tomography/computed tomography scanning showed multiple hyper-metabolic foci in the pelvic cavity and the spleen. The pathological diagnosis of the biopsy specimen was consistent with diffuse large B-cell lymphoma.

**Conclusion:**

This is the first report of pathologically confirmed case of CNS involvement of systemic diffuse large B-cell lymphoma with multifocal silent hemosiderin depositions detected by T2*-weighted gradient-echo magnetic resonance imaging. Even though uncommon, our report offers an insight that CNS lymphoma could present with multifocal silent hemosiderin depositions on T2*-weighted gradient-echo magnetic resonance imaging. Further studies were expected for exploring the association between this radiologic feature and systemic lymphoma and their underlying mechanisms.

## Background

Systemic non-Hodgkin lymphoma (NHL) may present variously in the central nervous system (CNS) involvement, of which parenchymal infiltration and leptomeningeal metastases are the most common types. The radiographic lesions tend to be non-hemorrhagic in the majority of cases. Intracranial hemorrhage in CNS lymphoma is extremely rare, and it was only reported in three case reports [1-3].

Hemorrhagic lesions in regular magnetic resonance imaging (MRI) are usually variable according to different clinical phases, while T2*-weighted gradient-echo magnetic resonance imaging (T2*-weighted MRI) is more sensitive and of particular use in detecting silent hemorrhage (including cerebral microbleeds and superficial siderosis etc.) as hypointense signals due to the deposition of paramagnetic hemosiderin or mineralization (e.g. calcium or iron) [4,5]. Herein, we present an unexpected radiographic feature on T2*-weighted MRI in a patient with CNS involvement of pathologically confirmed systemic diffuse large B-cell lymphoma.

## Case presentation

A 56-year-old woman presented with lower extremities weakness and progressive cognitive decline for four months. Initial laboratory studies revealed a mild anemia (hemoglobin level of 90 g/L). Erythrocyte sedimentation rate (44 mm/h) and serum C-reactive protein levels (50.71 mg/L) as well as serum lactic dehydrogenase (867 U/L) were markedly elevated. The investigation of cerebrospinal fluid (CSF) revealed no pleocytosis but a slightly elevated protein concentration (0.97 g/L). Brain MRI demonstrated multiple lesions with hypointensities on T1-weighted images and hyperintensities on T2-weighted images and fluid attenuated inversion recovery (FLAIR) in the bilateral periventricular areas, centrum semiovale and frontal lobes. She was then transferred to our hospital for further management.

On admission, neurological examination showed cognitive deficits in orientation, memory, calculation and language with a Mini-Mental State Exam score of 8/30 points. The muscle force was graded as 4/normally 5 for bilateral lower extremities with positive left Babinski sign.

While the serologic tests of treponemapallidum particle agglutination assay (TPPA) and fluorescent treponemal antibody absorption (FTA-ABS) IgG for syphilis were positive, the same tests in CSF were nonreactive plus the absence of relevant clinical manifestation, suggesting the diagnosis of neuro-syphilis unwarranted. The investigation of chemistry, cytology--particularly for neoplastic and atypical cells and other screening tests for bacteria, fungi and viruses in CSF, were negative. The serologic assay for human immunodeficiency virus was negative.

**Figure 1:**
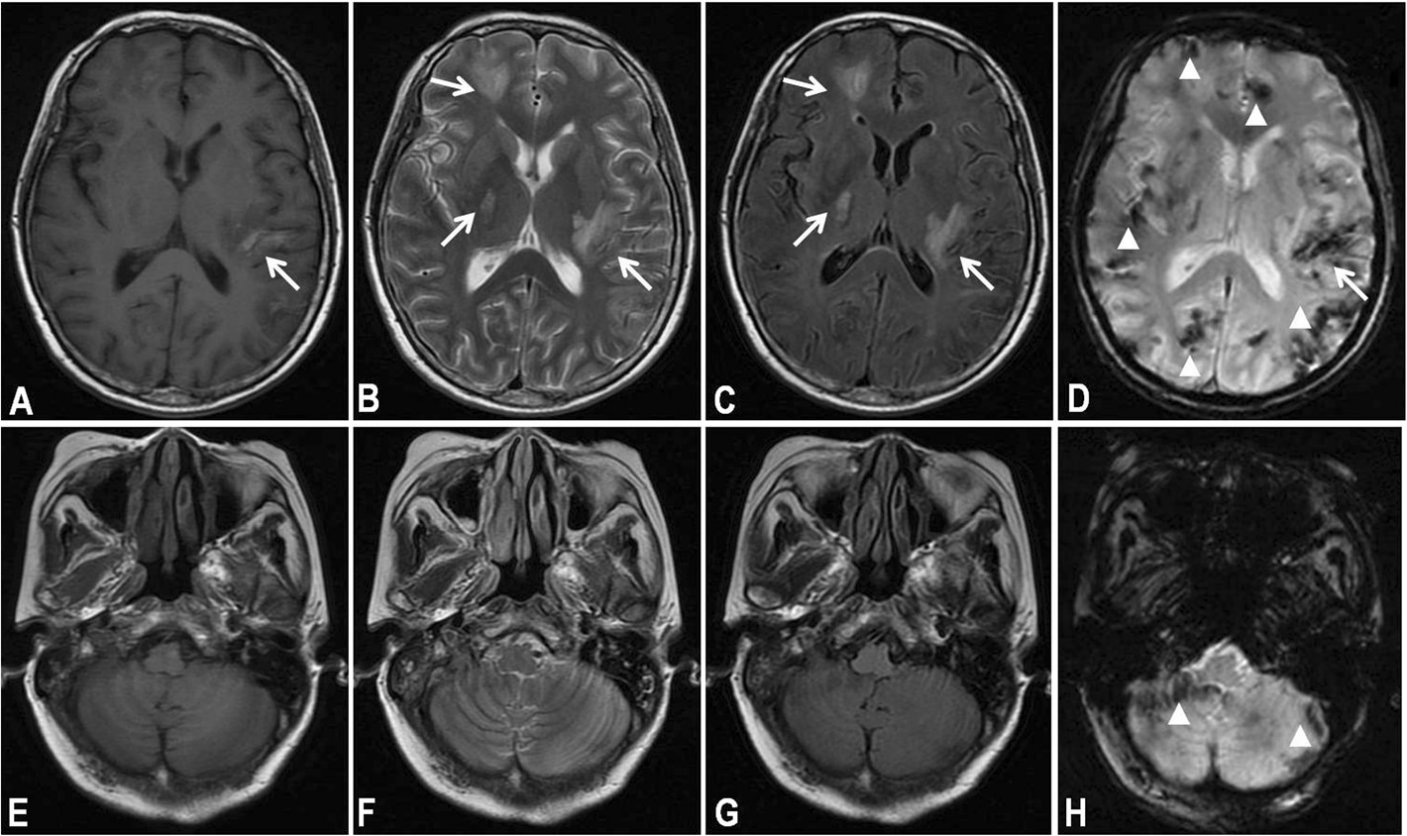
**Axial brain magnetic resonance imaging including supratentorial and infratentorial regions after admission. (A)** & **(E)** T1-weighted imaging demonstrated a hypointense area with focal hyperintensity in the left temporal lobe (arrow), suggesting newly patchy hemorrhage within the lesion. **(B)** & **(F)** T2-weighted imaging demonstrated multiple hyperintense lesions only involving the supratentorial region (arrows). **(C)** & **(G)** Fluid attenuated inversion recovery (FLAIR) showed multiple hyperintense lesions only involving the supratentorial region (arrows). **(D)** & **(H)** T2*-weighted MRI demonstrated diffuse hypointensities (arrowheads), indicating multifocal hemosiderin depositions in both supratentorial and infratentorial regions, which were not compatible with the hemorrhagic lesions visualized on T1-weighted images.

T1-weighted MRI demonstrated a hypointense area with focal hyperintensity in the left temporal lobe, suggesting newly patchy hemorrhage within the lesion. T2-weighted MRI and FLAIR showed no changes compared with the previous images (not shown). Part of lesions showed irregular spotty enhancement after contrast, together with the enhancement of adjacent leptomeninges. T2*-weighted MRI demonstrated multiple hypointensities in the right parietal lobe, bilateral basal ganglia and cerebellum (Figure 1), indicating hemosiderin depositions, however, the locations were not compatible with the hemorrhagic lesions visualized on T1-weighted images. Given the discrepancy between the lesions on T1-weighted and T2*-weighted MRI, silent hemorrhage caused by malignancy was suspected. The whole body positron emission tomography/computed tomography (PET/CT) scanning revealed multiple hypermetabolic foci in pelvic cavity and the spleen, indicating malignant lymphoma. Therefore, computed tomography-guided aspiration biopsy of the retroperitoneal mass was performed. Microscopic findings showed a diffuse growth pattern of atypical lymphoid cells with CD20 positive on immunohistochemistry, consistent with diffuse large B-cell lymphoma. The patient declined chemo-radiotherapy, and died of tumor progression four months later.

## Discussion

Multifocal hemosiderin depositions caused by chronic hemorrhage have not yet been identified in patients with CNS involvement of systemic lymphoma [6]. Even in primary CNS lymphoma, hemorrhagic lesions are extremely rare in immunocompetent patient [7]. Since 1990 to 2013, only three cases of intracranial hemorrhage were reported in primary CNS lymphoma. Rubenstein et al. [3] for the first time reported intracranial hemorrhage in a patient with primary CNS lymphoma. The intra-tumoral hemorrhage appeared hypointense on multiplanar gradient-refocused image, due to magnetic susceptibility effect caused by the blood-breakdown products. Kimura et al. [1] described a patient with primary CNS lymphoma with cortical laminar hemorrhage in the frontal cortex, showing hypointensity in the cortex on T2*-weighted MRI. The biopsy specimen revealed diffuse large B-cell lymphoma with hemosiderin depositions. Different from these two studies, in our case, the multifocal hypointensities indicating hemosiderin depositions detected by T2*-weighted MRI located mostly adjacent to the cortex, which were not compatible with the hemorrhagic lesions visualized on T1-weighted MRI. To our knowledge, this is the first report of pathologically confirmed case of secondary CNS lymphoma with multifocal hemosiderin depositions on T2*-weighted MRI. Importantly, physicians should be alert to the diagnosis of systemic lymphoma when confronting this unusual radiographic feature.

Conventional MRI is commonly used for the detection of CNS lymphoma, on which lymphoma usually appears as non-hemorrhagic lesion. However, T2*-weighted MRI, sensitive to cerebral hemorrhage, is not routinely employed. This may result in undetected silent hemorrhage or microhemorrhage in some patients. In our case, T2*-weighted MRI, played a very important role in providing a valuable clue leading to the diagnosis of CNS lymphoma, taken together with the information on conventional MRI. Even though uncommon, our report offers an insight that CNS involvement of systemic diffuse large B-cell lymphoma could present with multifocal silent hemosiderin depositions on T2*-weighted MRI. The conventional MRI including non-enhanced and contrast-enhanced MRI is insufficient for detecting the lesions of CNS lymphoma with diverse properties. T2*-weighted MRI could be taken into account when confronted with the patients suspected of having CNS lymphoma. We expect further studies exploring the association between this radiologic feature and systemic lymphoma and their underlying mechanisms in order to better understand and manage this life-threatening disease.

Cerebral amyloid angiopathy (CAA) is ranked highly in the differential diagnosis of multifocal hemosiderin depositions among elderly patients [8]. The most common clinical manifestation of spontaneous lobar hemorrhage and radiographic feature of restricted location in lobar, cortical, or corticosubcortical regions were not demonstrated in this case. Thus, the possible diagnosis of CAA was less likely considered.

## Conclusion

This is the first report of pathologically confirmed case of CNS involvement of systemic diffuse large B-cell lymphoma with multifocal silent hemosiderin depositions detected by T2*-weighted MRI. Even though uncommon, our report offers an insight that CNS lymphoma could present with multifocal silent hemosiderin depositions on T2*-weighted MRI. Further studies were expected for exploring the association between this radiologic feature and systemic lymphoma and their underlying mechanisms.

## Consent

Written informed consent was obtained from the patient for publication of this Case report and any accompanying images. A copy of the written consent is available for review by the Editor-in-Chief of this journal.
